# Prize of One Hundred Pounds for an Essay on Hydrophobia

**Published:** 1879-01

**Authors:** 


Prize of One Hundred Pounds for an Essay on Hydrophobia: Its Nature, Prevention and Treatment, Offered by V. F. Benett Stanford, Esq., M.P., to be Awarded by this Royal College of Physicians of London.—Conditions under which the above prize is to be competed for:

1. The essay must be in English, or accompanied by an English translation.

2. The essay must be delivered to the College on or before January 1, 1880.

3. Each essay to be accompanied by a sealed envelope containing the name and address of the author and bearing a motto on the outside, the same motto to be inscribed on the essay.

4. The essay may be the joint production of two or more authors.

5. The essay, if not published by the author within a year, to become the property of the College.

6. The prize not to be awarded unless an essay of sufficient merit be presented.

The questions which are thought by the College specially to require investigation are:

1. The origin and history of outbreaks of rabies, particularly in the United Kingdom and its dependencies.

2. The best mode of prevention of rabies.

3. The characteristics of rabies during life, and the anatomical and chemical changes which are associated with the disease in its successive stages, particularly in its commencement.

4. The origin of hydrophobia in man.

5. The chemical and anatomical morbid changes observed in the subjects of the disease, with special reference to those having their seat in the organ of the nervous system and in the salivary glands.

6. The symptoms of the disease, particularly of its early stage, as illustrated in well observed cases.

7. The diagnosis of the disease in doubtful cases, from conditions more or less resembling it.

8. The alleged prolonged latency of the malady.

9. The efficacy of the various remedies and modes of preventing the disease which have been proposed, and what plan of treatment, whether prophylactic or curative, it would be most desirable to recommend for future trial.—*The New Orleans Medical and Surgical Journal.*

